# Supplementary material for: A complex genomic locus drives mtDNA replicase POLG expression to its disease‐related nervous system regions
Source: EMBO Mol Med. 2017 Nov 6;10(1):13–21. doi: 10.15252/emmm.201707993 (PMC5760859; doi:10.15252/emmm.201707993)
Supplement: Supplementary file 1 — Appendix [file EMMM-10-13-s001.pdf]

**Table of Contents**

Supplementary Figure Legends

Appendix Figure S1. Enhancer element 1, 2 and 3 founder lines.

Appendix Figure S2. mRNA expression of *Polg* and *Ai854517* in liver.

Appendix Table S1. Transcription factor binding sites within *POLG* enhancers.

Appendix Table S2. Primer sequences.

## Supplementary Figure Legends

**Appendix Figure S1. Enhancer element 1, 2 and 3 founder lines.** (A) Three independent founder lines of transgenic mouse lines carrying enhancer elements 1, 2 or 3 with *lacZ* marker gene. Blue color shows *lacZ* expression.

**Appendix Figure S2. mRNA expressions of *Polg* and *Ai854517* in liver.** (A) mRNA expressions of *Polg* and *Ai854517* in C57BL/6 male mouse liver and cerebellum. n = 4 (B) RNA expressions of MIR9, *NR2E2*, *LDLDRAP1* and *MTHFD2* in HEK293 untransfected controls and in cells transfected with pre-MIR9 or scrambled RNA. Shown is mean with standard error of the mean. Abbreviations: AU; arbitrary units.

**Appendix Table S1.** Transcription factor binding sites within *POLG* enhancer elements 1, 2 and 3.

**Appendix Table S2.** Primer sequences. The added restriction sites used for cloning are shown in bold in the primer sequence.

Appendix Figure S1.

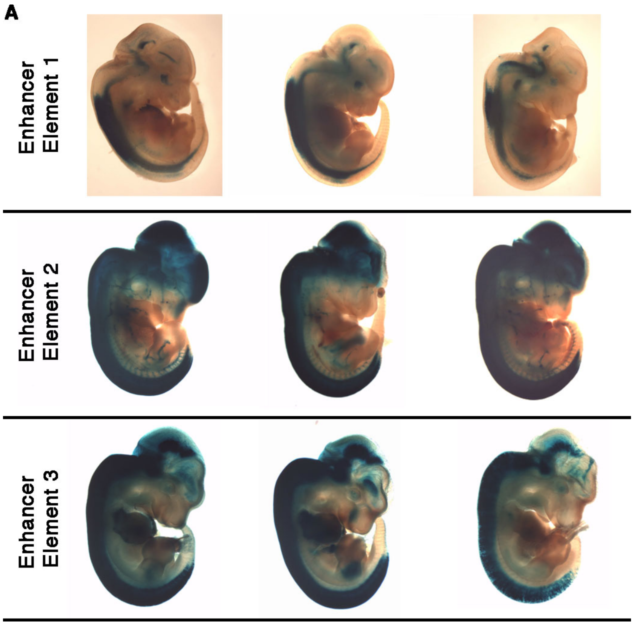

Appendix Figure S2.

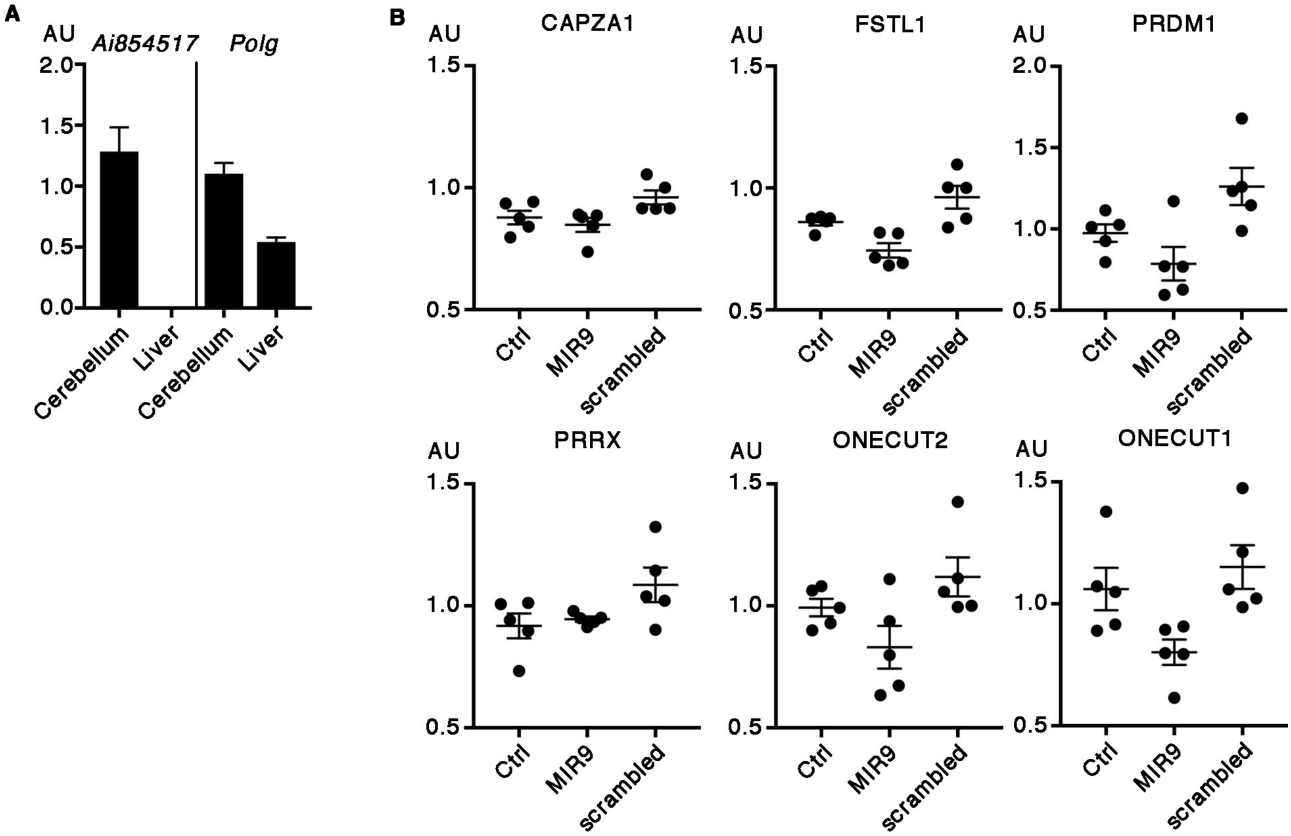

Appendix Table S1.

| Transcription factor binding sites in *POLG* enhancer elements | | |
|---|---|---|
| | | |
| **EE1** | **EE2** | **EE3** |
| C-REL | Agamous | Su(H) |
| Dof2 | MZF1-4 | Hunchback |
| Dof2 | Tal1beta-E47S | jsmad5 |
| S8 | jsmad5 | SP1 |
| HNF-3beta | Thing1-E47 | SPI-1 |
| TBP | RORalpha-2 | MZF1-4 |
| RORalpha-1 | HMG-IY | NF-kB |
| NRF-2 | deltaEF1 | MZF1-4 |
| gli2 | jsmad5 | Bsap |
| Yin-Yang | Broadc3 | jci |
| SQUA | MZF1-4 | AHR_ARNT |
| jsmad5 | NXF_ARNT | Dof3 |
| Su(H) | jtcf4 | S8 |
| S8 | AGL3 | jgli |
| SRY | jsmad5 | Athb-1 |
| NRF-2 | jsmad5 | Sox-5 |
| Myf | Dorsal1 | NF-kB |
| Su(H) | SOX17 | SP1 |
| jsmad5 | Gfi | |
| Yin-Yang | SOX17 | |
| Androgen | SOX17 | |
| HMG-IY | SP1 | |
| MEF2 | SP1 | |
| NF-Y | FREAC-4 | |
| SPI-1 | Myf | |
| MZF5-13 | | |
| SRF | | |
| jtcf4 | | |
| jsmad5 | | |
| MZF1-4 | | |
| Hunchback | | |
| Dof2 | | |

Appendix Table S2

| Gene | Organism | Forward primer (5' to 3') | Reverse primer (5' to 3') | Application |
|---|---|---|---|---|
| *Actb* | mouse | ATGCTCCCCGGGCTGTAT | CATAGGAGTCCTTCTGACCCATTC | QPCR |
| *Polg* | mouse | TCATGGCACTGACCTGGGATG | TGTCAGTGAGTAAGAACTCCT | QPCR |
| *Ai854517* | mouse | CAGGAGACTGAATGGGGAAA | GTCCGGTGGTGTCTCTGAAT | QPCR |
| *ACTB* | human | CCTGGCACCCAGCACAAT | GGGCCGGACTCGTCATAC | QPCR |
| *POLG* | human | TTTCCTTTGACCGAGCTCAT | GCCTCTTCTGGCTTTCCTCT | QPCR |
| *LINC00925* | human | CTGACCCCAAAGAGATCCTG | CAGGAGAAGCCCAGCTAAAA | QPCR |
| *MTHFD2* | human | GATCAAGGAAGGAGCAGCAG | TTCAAGCCTCAGCACCTTTT | QPCR |
| *LDLRAP1* | human | CGGGGAATTATCCTGACAGA | CAAACACCTTGTCGTGCATC | QPCR |
| *FSTL1* | human | TGGCAAGACCTACCTCAACC | CCAGCCATCTGGAATGATCT | QPCR |
| *CAPZA1* | human | ATGGATCAGTTCACGCCTGT | GGGGTCACTTGCTTCTTTCC | QPCR |
| *PRDM1* | human | GCCACCAACAGTGAAGAGGT | TTCCTGTTGGCGTTCTTAGG | QPCR |
| *PRRX* | human | AGCAGCGAAGGAATAGGACA | ACTTGGCTCTTCGGTTCTGA | QPCR |
| *ONECUT1* | human | AACCCTGGAGCAAACTCAAA | TGGATGGACGCTTATTTTCC | QPCR |
| *ONECUT2* | human | AAATCTGGCAGGGAGACCTT | CTGCTGGGAAATGGTGATCT | QPCR |
| *NR2E1* | human | TGCCTGTCTAAAATGCATCG | GCTGAGCCTCATCTTGAAGG | QPCR |
| *Ai854517* | mouse | **CTCGAG**CACATGCCTAGACAGGAGCA | **GAATTC**CCCATTCAGTCTCCTGCATT | In situ |
| *Polg* | mouse | **CTCGAG**ACCTGGCTATGTCCTTGTGG | **GAATTC**ACCTGGTCAGGAGATTGGTG | In situ |
| Enhancer 1 | mouse | TGAC**AAGCTT**CAGATGCACAACCATGGACT | GCAG**GCATGC**CACTGACCTAAGTGTGACTCAGC | Cloning |
| Enhancer 2 | mouse | TGAC**CCTGCAGG**AGACTTCAGCGTGTGCAGTG | GCAG**GTCGAC**TGCTAAGCCTGAGGTGAGAAA | Cloning |
| Enhancer 3 | mouse | TGAC**AAGCTT**GCTGGGTCTACATCCACCAC | GCAG**GCATGC**CGAAGTGGAGTGGGTTGG | Cloning |
| *Polg* promoter | mouse | AGT**GGATCC**GCGGTCCATAGGTGACAAAT | AGT**GGATCC**CCAATAAGAGCGCCGCCAAT | Cloning |
